# The causal relationship between matrix metalloproteinase-3 and fibromyalgia: A two-sample Mendelian randomization analysis

**DOI:** 10.1097/MD.0000000000044420

**Published:** 2025-09-12

**Authors:** Honglin Wang, Hong Zhou, Yifan Cai, Zhewei Ye

**Affiliations:** a Department of Orthopedics Surgery, Union Hospital, Tongji Medical College, Huazhong University of Science and Technology, Wuhan, China; b Intelligent Medical Laboratory, Union Hospital, Tongji Medical College, Huazhong University of Science and Technology, Wuhan, China; c Department of Orthopedics, Fuzhou University Affiliated Provincial Hospital, Fuzhou, China; d Cancer Center, Union Hospital, Tongji Medical College, Huazhong University of Science and Technology, Wuhan, China.

**Keywords:** causal relationship, fibromyalgia, genetic epidemiology, matrix metalloproteinase-3, Mendelian randomization

## Abstract

Fibromyalgia is a chronic pain syndrome with incompletely understood pathogenesis. Matrix metalloproteinase-3 (MMP-3), a key enzyme involved in inflammation and tissue remodeling, has not been genetically validated for its causal relationship with fibromyalgia. This study aims to explore the causal association between MMP-3 and fibromyalgia using Mendelian randomization (MR) methods. Genome-wide association study (GWAS) data for MMP-3 levels (n = 21,758) were obtained from the IEU Open GWAS database, and GWAS data for fibromyalgia were extracted from public databases (n = 361,194). Inverse variance weighting (IVW), MR-Egger, weighted median, and weighted mode were used to evaluate causal effects. Heterogeneity tests, horizontal pleiotropy tests, and leave-one-out sensitivity analyses were performed to verify the robustness of the results. IVW analysis showed that per 1 standard deviation increase in MMP-3 level was associated with a 0.098% increase in fibromyalgia risk (odds ratio [OR] = 1.00098, 95% confidence interval = 1.00014–1.00182, *P* = .0225). MR-Egger (OR = 1.00050, *P* = .5183) and weighted median (OR = 1.00081, *P* = .0982) results were consistent with IVW but not statistically significant. Sensitivity analyses showed no significant heterogeneity (Cochran *Q* test, *P* > .05) or horizontal pleiotropy (MR-Egger intercept, *P* = .4391), and leave-one-out analysis indicated that individual single nucleotide polymorphisms did not affect the overall results. This study supports a potential causal association between MMP-3 and fibromyalgia through genetic evidence, suggesting that MMP-3 may be involved in the pathogenesis of fibromyalgia.

## 1. Introduction

Fibromyalgia is an idiopathic, common, and complex syndrome characterized by long-term, widespread, and symmetrical nonarticular musculoskeletal pain.^[1]^ The global prevalence is ~2% to 5%, with a female predominance.^[2]^ However, current treatments are limited to relieving pain and improving quality of life, including analgesic drug therapy, physical therapy, psychological therapy, etc.^[3–5]^ Its pathophysiology is complex, involving central sensitization, immune dysfunction, and neuroendocrine abnormalities, but the genetic and molecular mechanisms remain unclear.^[6]^ Among many potential factors, matrix metalloproteinase-3 (MMP-3) has attracted attention for its possible involvement in the pathogenesis of fibromyalgia.

The matrix metalloproteinase family is a group of zinc-dependent endopeptidases. MMP-3 belongs to the stromelysin family of MMPs and exhibits broad substrate specificity, making it a key participant in degrading the extracellular matrix (ECM).^[7]^ Many studies have found associations between MMP-3 levels and multiple aspects of fibromyalgia, such as pain sensitivity and disease activity. For example, in fibromyalgia patients, levels of MMP-3 in cerebrospinal fluid after exercise are negatively correlated with pain thresholds, implying that MMP-3 may play a promoting role in pain perception in fibromyalgia.^[8]^ Additionally, serum MMP-3 levels are elevated in inflammatory rheumatic diseases, especially polymyalgia rheumatica.^[9]^ However, these studies are susceptible to confounding factors and cannot clarify the directionality, which hinders the exact determination of the causal relationship between MMP-3 and fibromyalgia.

Mendelian randomization (MR) is a causal inference method based on genetic variations. By using large-scale genome-wide association study (GWAS) data and genetic variations strongly associated with exposure factors, MR infers the potential causal relationship between exposure and outcome, effectively reducing the influence of confounding factors, reverse causality, and the limitations of observational studies.^[10]^ This study first uses 2-sample MR to analyze the causal association between MMP-3 levels and fibromyalgia, aiming to provide genetic evidence for the pathogenesis and potential therapeutic targets of fibromyalgia.

## 2. Materials and methods

### 2.1. Data source

Exposure data: GWAS data for MMP-3 levels were obtained from the study by Folkersen et al^[11]^ (IEU Open GWAS ID: ebi-a-GCST90012027), including 21,758 individuals of European descent, identifying 13,057,986 single nucleotide polymorphisms (SNPs) associated with MMP-3.

Outcome data: GWAS data for fibromyalgia were obtained from public databases (ID: ukb-d-FIBRO_COMORB), including 361,194 individuals of European descent, with 10,692,533 SNPs associated with fibromyalgia.

This study used publicly available GWAS data which were previously approved by the respective institutional review boards of the data-providing institutions. Since this study is a secondary analysis of deidentified public data, additional ethical approval was not required. Informed consent was not applicable as the data were anonymized and publicly accessible.

### 2.2. Instrumental variable (IV) selection

SNPs significantly associated with MMP-3 levels (*P* < 5 × 10⁻^8^) were screened, and independent SNPs were retained through linkage disequilibrium analysis (*r*^2^ < 0.001, clumping window size = 10,000 kb). Finally, 7 SNPs were included as IVs. Meanwhile, the *F* statistic for each SNP was calculated to assess the strength of the IV. These SNPs all meet the criteria for strong IVs (with *F* > 10, ranging from 31.09 to 1376.10), thus excluding weak IV bias (Table 1).

**Table 1:** Information on the final screening of matrix metalloproteinase-3 SNPs from GWAS data (n = 7).

SNP	Effect_Allele	Other_Allele	*β*	SE	*P*	*F*
rs11668189	A	C	−0.0557	0.01	3.2 × 10⁻^8^	31.09
rs2186789	C	A	0.1337	0.011	5.6 × 10⁻^10^	139.48
rs2267373	T	C	0.0570	0.0096	2.1 × 10⁻^7^	34.46
rs3020919	T	C	0.2572	0.0106	8.9 × 10⁻^15^	539.42
rs471994	A	G	0.3597	0.0094	1.2 × 10⁻^22^	1376.10
rs495041	T	C	0.0722	0.0131	4.3 × 10⁻^6^	32.78
rs72971614	G	T	0.1121	0.0141	2.7 × 10⁻^9^	63.27

GWAS = genome-wide association study, SNP = single nucleotide polymorphism.

### 2.3. MR analysis

The following MR methods were mainly used to estimate causal effects. Inverse variance weighting (IVW): integrates the Wald ratio of individual SNPs, assuming all IVs are valid and free of horizontal pleiotropy, providing the most precise causal effect estimate with the highest statistical power. MR-Egger: introduces an intercept term based on the IVW model to account for potential horizontal pleiotropy, providing a consistent estimate of the causal effect even in the presence of pleiotropy. Weighted median: weights based on the median of all IV effects, obtaining a consistent estimate even in the presence of pleiotropy or weak IVs, as long as valid IVs account for ≥50%. Weighted mode method: suitable for scenarios with a large number of invalid IVs, inferring causal relationships through the majority-consistent effect direction.

Sensitivity analysis: Cochran *Q* test was used to assess heterogeneity, detecting heterogeneity in effects among IVs to determine potential confounding or method bias. MR-Egger intercept test evaluated horizontal pleiotropy to assess whether IVs affect outcomes through nonexposure pathways. MR-PRESSO (MR pleiotropy residual sum and outlier) analysis was used to detect horizontal pleiotropy and outliers in IVs. Additionally, leave-one-out method was used to exclude each SNP one by one, recalculate causal effects, analyze the impact of individual SNPs on results, and observe the stability of effect estimates.

### 2.4. Statistical analysis

R software v4.4.2 (R Foundation, Vienna, Austria) packages including 2-sample MR, MR, and MR-PRESSO were used for analysis. Odds ratios (ORs) and their 95% confidence intervals (CIs) were used to represent results. In the main MR analysis, *P* < .05 was considered statistically significant. In sensitivity analyses, Cochran *Q* test *P* < .05 indicated significant heterogeneity, MR-Egger intercept test *P* < .05 indicated directional pleiotropy, and MR-PRESSO global test *P* < .05 indicated pleiotropy.

## 3. Results

### 3.1. Causal effect estimation

Four MR analysis methods all suggested a positive correlation trend between MMP-3 levels and fibromyalgia. The IVW analysis showed a positive correlation between MMP-3 levels and fibromyalgia (OR = 1.00098, 95% CI = 1.00014–1.00182, *P* = .0225), indicating that an increase in MMP-3 may increase the risk of fibromyalgia. Other methods, though not significant, showed consistent effect directions, supporting the robustness of the causal association (Table 2).

**Table 2:** MR analysis results between matrix metalloproteinase-3 and fibromyalgia (*P* < .05).

Method	nSNPs	*β*	SE	OR	95% CI	*P*
IVW	7	0.00098	0.00043	1.00098	1.00014–1.00182	.0225
MR-Egger	7	0.00050	0.00072	1.00050	0.99910–1.00190	.5183
Weighted median	7	0.00081	0.00049	1.00081	0.99984–1.00178	.0982
Weighted mode	7	0.00062	0.00048	1.00062	0.99969–1.00156	.2400

All data are accurate to 4 decimal places.

CI = confidence interval, IVW = inverse variance weighted, MR = Mendelian randomization, OR = odds ratio, SNP = single nucleotide polymorphism.

**Figure 1.**
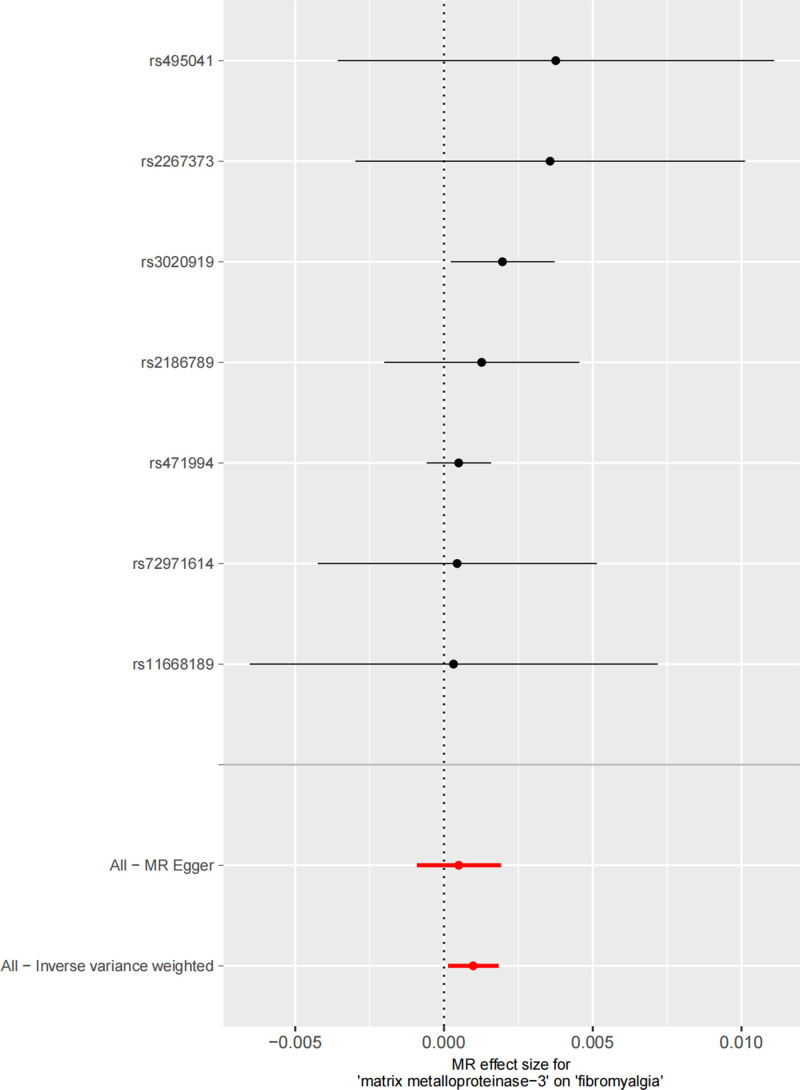
Forest plot for matrix metalloproteinase-3 and fibromyalgia (*P* < .05). MR = Mendelian randomization.

**Figure 2.**
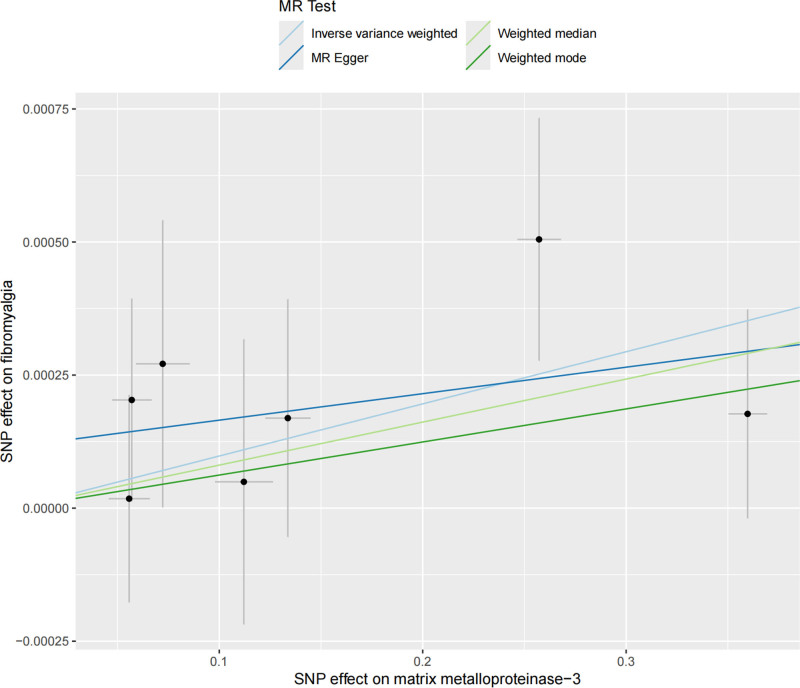
Scatter plots for matrix metalloproteinase-3 and fibromyalgia. MR = Mendelian randomization, SNP = single nucleotide polymorphism.

The forest plot intuitively shows the causal effects of individual SNPs and the combined results: the horizontal bars represent the OR and 95% CI of individual SNPs, and the diamond represents the combined effect of IVW. Most SNP effect estimates lie to the right of the midline (OR > 1), supporting that increased MMP-3 is associated with increased fibromyalgia risk (Fig. 1). Genetic association between MMP-3 and fibromyalgia: the horizontal axis is the effect of SNPs on MMP-3, and the vertical axis is the effect of SNPs on fibromyalgia. Solid points represent the combined effects of different MR methods (IVW, MR-Egger, etc), and consistent arrow directions indicate the robust direction of the causal association (Fig. 2).

### 3.2. Sensitivity analysis

**Figure 3.**
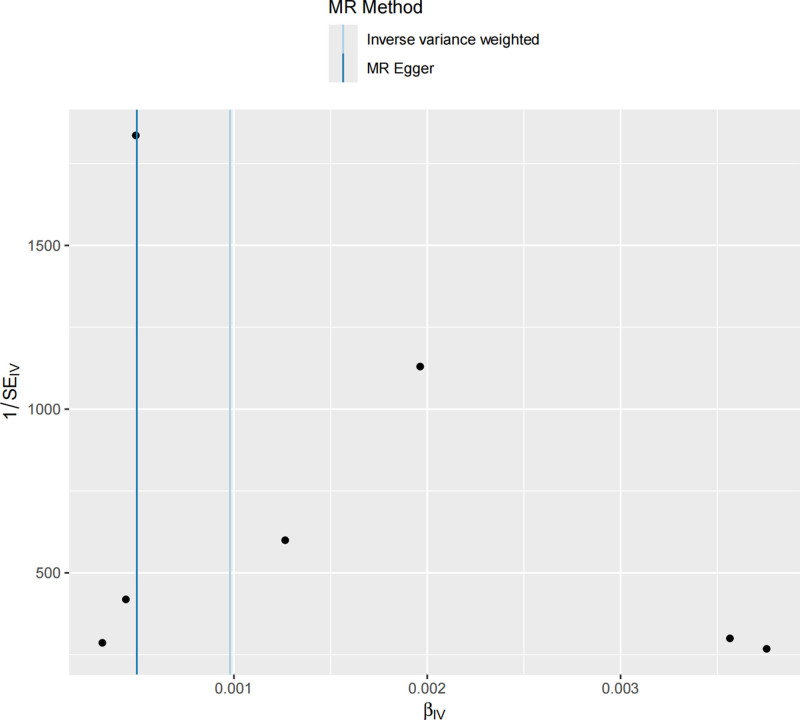
Funnel plot of MR analysis for matrix metalloproteinase-3 and fibromyalgia. MR = Mendelian randomization.

Heterogeneity test: Cochran *Q* test showed no significant heterogeneity (IVW: *Q* = 3.31, *P* = .770), indicating consistent effects among SNPs. Horizontal pleiotropy: The MR-Egger intercept was 0.00012 (*P* = .4391), suggesting no significant horizontal pleiotropy. The MR-PRESSO global test also found no systematic pleiotropy (*P* = .6041) (Table 3). The points in the funnel plot represent the effect estimates of each SNP and are symmetrically distributed on both sides of the midline, indicating no significant bias or heterogeneity (Fig. 3).

**Table 3:** Sensitivity analysis of the MR analysis results between matrix metalloproteinase-3 and fibromyalgia (*P* > .05).

Exposure	Outcome	Cochran *Q* test (IVW)		MR-Egger		MR-PRESSO	
		*Q*	*P*	Intercept value	*P*	*T*-stat	*P*
Matrix metalloproteinase-3	Fibromyalgia	2.60	.761	0.00012	.4391	3.0740	.6041

IVW = inverse variance weighted, MR = Mendelian randomization, MR-PRESSO = MR pleiotropy residual sum and outlier.

**Figure 4.**
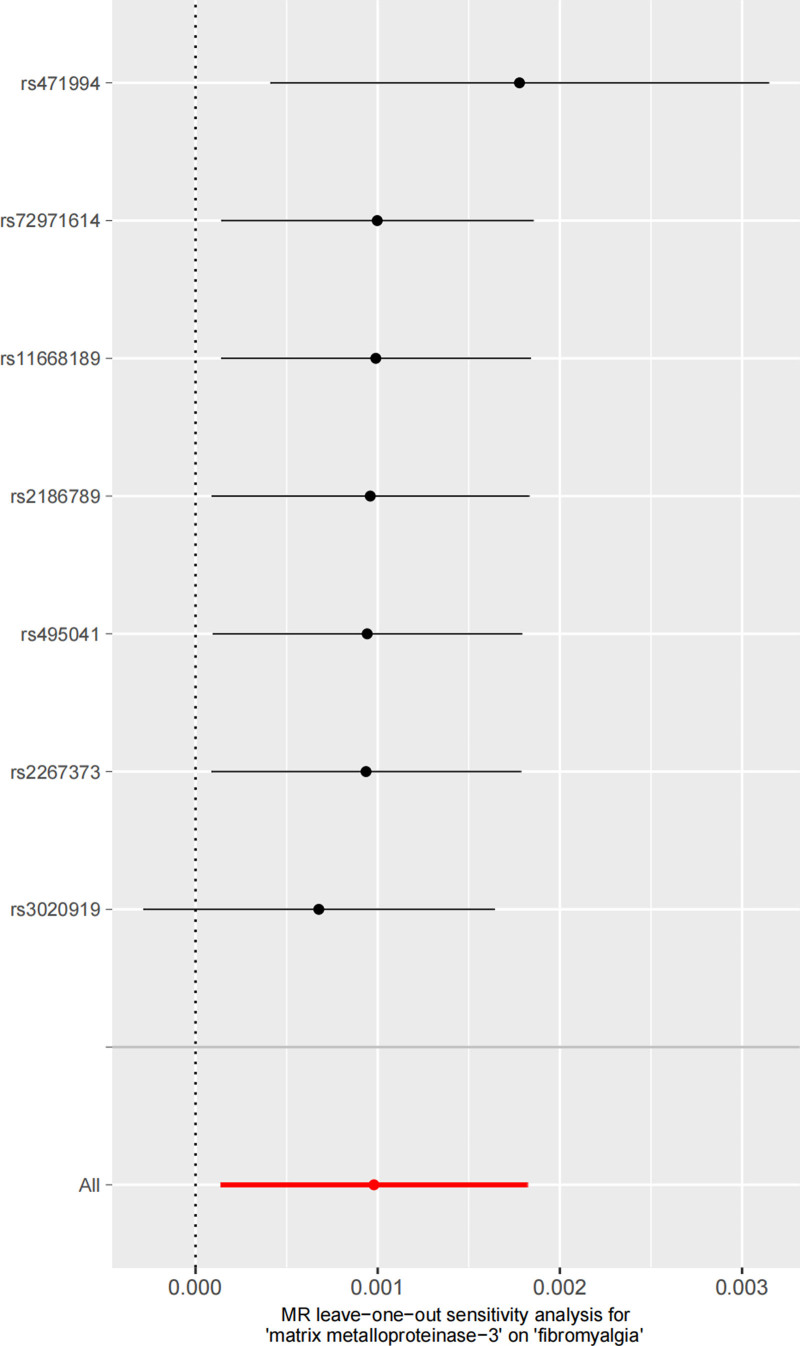
The leave-one-out analysis for matrix metalloproteinase-3 and fibromyalgia. MR = Mendelian randomization.

Additionally, we analyzed the impact of individual SNPs on the overall causal estimation through leave-one-out analysis. As shown in the forest plot of Figure 4, each row represents the IVW effect estimate after excluding 1 SNP, and the diamond represents the overall result. The results showed that after removing any single SNP, the OR value remained between 1.0006 and 1.0018, indicating that the results were not affected by a single genetic variation and had good robustness.

## 4. Discussion

Many orthopedic and joint diseases with unclear etiology are associated with excessive inflammatory responses,^[12]^ such as osteoarthritis^[13]^ and rheumatoid arthritis.^[14]^ Both osteoarthritis and rheumatoid arthritis are accompanied by inflammatory responses and upregulation of MMP-3.^[15,16]^ The pathogenesis of fibromyalgia is also closely related to inflammation.

Other observational studies have linked MMP-3 levels to pain sensitivity in fibromyalgia patients (e.g., a negative correlation between cerebrospinal fluid MMP-3 and pain thresholds after exercise).^[8]^ However, these studies are limited by confounding factors and cannot establish the causal direction. This study uses genetic variations (SNP) as IVs to reduce reverse causality and confounding bias, providing the first genetic evidence for the potential role of MMP-3 in the pathogenesis of fibromyalgia. This study reveals a potential causal association between MMP-3 levels and fibromyalgia. IVW analysis shows that per 1 standard deviation increase in MMP-3 levels is associated with a 0.098% increase in fibromyalgia risk (OR = 1.00098, 95% CI = 1.00014–1.00182, *P* = .0225). Although some MR methods (such as MR-Egger) did not reach statistical significance, which may be related to small sample size or differences in method assumptions, all methods showed consistent effect directions, and sensitivity analyses supported the robustness of the results. The limitations of this study include: the sample is limited to European populations, and extrapolation to other races requires caution; the GWAS data of fibromyalgia did not provide gender/age stratification; residual pleiotropy cannot be completely excluded, although the intercept test showed no significant horizontal pleiotropy; and genetic variations in MMP-3 may only explain part of the variation in serum levels, with the possibility of residual weak IV bias.

It should be noted that this study relies on the MR assumption that outcomes are affected by IVs only through the exposure factor. Fibromyalgia is influenced by both genetic and environmental factors (such as stress and infection). Although sensitivity analyses ruled out a significant horizontal pleiotropy, mechanistic studies are still needed to validate whether MMP-3 is involved in fibromyalgia pathogenesis under different environmental exposures.

This study has clinical guiding significance. First, MMP-3 may serve as a biomarker for fibromyalgia risk assessment or treatment monitoring. Additionally, targeting MMP-3 or its pathway may become a novel therapeutic strategy. If the causal association between MMP-3 and fibromyalgia is validated in larger samples, it could guide early intervention strategies in high-risk populations.

As a zinc-dependent protease, MMP-3 degrades the ECM, promotes the release of inflammatory factors, and participates in neuropathic pain and central sensitization.^[17–19]^ Existing studies have shown that the development of MMP inhibitors has advanced from first-generation broad-spectrum inhibitors (e.g., batimastat) to second-generation specific inhibitors. For example, tofacitinib significantly reduces the expression of MMP-3 in chondrocytes and diminishes interleukin-6 (IL-6)-induced inflammatory responses by inhibiting the JAK-STAT pathway.^[20]^ This provides a reference for the treatment of fibromyalgia. Given the presence of central sensitization and neuroinflammation in fibromyalgia patients, specific MMP-3 inhibitors may exert effects through the following pathways: inhibiting neuroinflammation: reducing the release of inflammatory factors such as IL-6 and TNF-α, and lowering the pain sensitivity of the central nervous system; stabilizing the blood-brain barrier: regulating ECM degradation to reduce abnormal pain signal transduction caused by increased blood-brain barrier permeability^[21]^; and modulating immune-neural crosstalk: blocking the binding of MMP-3 to cytokines (e.g., IL-6) to improve immune dysfunction and neuroendocrine abnormalities associated with fibromyalgia.^[8]^ It should be noted that MMP-3 is involved in tissue repair under physiological conditions, and excessive inhibition may lead to side effects such as delayed wound healing. Therefore, developing inhibitors with both specificity and tissue targeting (such as local delivery systems) is a key direction, which provides new ideas for the precise treatment of fibromyalgia.^[7,22]^

Regarding the generalizability of this study, it is important to note that the GWAS data were from European populations. The prevalence of fibromyalgia and genetic backgrounds vary across ethnicities. The frequencies and effects of MMP-3-related SNPs may differ in non-European populations, necessitating cross-ethnic MR studies or local GWAS validation. Furthermore, the study did not include special populations (such as children, the elderly, or patients with comorbidities). The pathogenesis of fibromyalgia may vary by age or comorbidities, so stratified analysis is needed in the future.

MMP-3 levels fluctuate with age and physiological states. This study used cross-sectional MMP-3 GWAS data, precluding inference of the temporal sequence between MMP-3 elevation and fibromyalgia onset. Longitudinal GWAS or dynamic MR analyses are needed to clarify whether MMP-3 levels in midlife or adolescence correlate with fibromyalgia risk. Moreover, the effects of acute MMP-3 elevation (such as during infection) may differ from those of chronic elevation on fibromyalgia. The results of this study reflect the association with genetically predicted long-term MMP-3 levels, rather than short-term fluctuations.

Concerning exposure levels, this study analyzed overall MMP-3 elevation but did not explore the effects of different exposure levels (mild/moderate/severe). The IVW result (a 0.098% risk increase per 1 standard deviation of MMP-3) suggests a weak dose–response relationship, which requires confirmation through larger samples or quantile MR analysis. Additionally, the association between MMP-3 and fibromyalgia may be nonlinear (such as a threshold effect), where both extremely low and high MMP-3 levels may increase the risk. This was not addressed in the current analysis and requires further investigation.

## 5. Conclusion

Based on 2-sample MR analysis, genetically predicted MMP-3 levels are positively correlated with fibromyalgia, supporting that MMP-3 may be involved in the pathogenesis of fibromyalgia. These findings provide a new perspective on the etiology of fibromyalgia and highlight the potential value of MMP-3 as a therapeutic target.

## Author contributions

**Conceptualization:** Honglin Wang.

**Data curation:** Honglin Wang, Yifan Cai.

**Formal analysis:** Honglin Wang, Yifan Cai.

**Funding acquisition:** Zhewei Ye.

**Investigation:** Honglin Wang, Yifan Cai.

**Methodology:** Honglin Wang, Hong Zhou.

**Project administration:** Zhewei Ye.

**Resources:** Hong Zhou.

**Software:** Hong Zhou, Yifan Cai.

**Supervision:** Zhewei Ye.

**Validation:** Yifan Cai.

**Visualization:** Yifan Cai.

**Writing – original draft:** Honglin Wang.

**Writing – review & editing:** Hong Zhou, Yifan Cai, Zhewei Ye.
